# *Notes from the Field:* Age Distribution of Patients with Laboratory-Detected Respiratory Syncytial Virus — Arizona, 2013–2017

**DOI:** 10.15585/mmwr.mm6808a4

**Published:** 2019-03-01

**Authors:** Rebecca Bridge, Shane Brady, Laura M. Erhart, Kenneth Komatsu

**Affiliations:** 1 Arizona Department of Health Services.

Respiratory syncytial virus (RSV)–positive laboratory detections have been reportable in Arizona since 2004 as part of the state’s passive infectious disease surveillance. All Arizona clinical laboratories are mandated to report*; however, some health care providers also report RSV detections, and surveillance includes both inpatient and outpatient facilities. A case is defined as a laboratory-positive result reported during the RSV season, which is from October to September in Arizona. During the 2016–17 season, the Arizona Department of Health Services noted a shift in age distribution of patients with reported detections. During the 2009–10 through 2012–13 seasons, >90% of reported cases each season were among children aged <5 years. In the 2016–17 season, the percentage of cases in children aged <5 years declined to 78%, whereas the percentage among adults aged ≥65 years increased from 1% in 2009–10 to 11% in 2016–17. Simultaneous with this observed change in age distribution, an overall increase in polymerase chain reaction (PCR) testing for RSV diagnosis and a decrease in antigen-based RSV testing has been reported in the United States (*1*,*2*). The Arizona Department of Health Services analyzed RSV surveillance data to investigate whether the observed shift in age distribution of patients with RSV reflected a change in RSV epidemiology or was related to changes in testing practices, including an increase in PCR use.

During four RSV seasons (2013–14 through 2016–17), approximately 3,000–5,000 cases were reported each season in Arizona. Reported laboratory tests were categorized as rapid antigen, PCR, or other (i.e., culture or immunofluorescence assays). The percentage of tests that could not be categorized ranged from 1% (2015–16 season) to 11% (2013–14).

All analyses were performed using SAS software (version 9.4, SAS Institute). Children aged <5 years accounted for a decreasing percentage of reported cases in each successive RSV season from 2013–14 to 2016–17 (89%, 84%, 82%, and 78%) (Table), while the percentage of cases in persons aged ≥65 years increased in each successive season (4%, 6%, 9%, and 11%) (chi-squared test for trend, p<0.001). Simultaneously, the percentage of positive test results by PCR increased 152%, from 21% of cases with a categorized test during 2013–14, to 53% during 2016–17 (p<0.001). Notably, although the percentage of cases with PCR testing increased among all age groups during this period, the largest percentage increase in reported cases was in patients aged ≥65 years. In addition, over the four RSV seasons, the percentage of reported PCR detections in patients aged ≥65 years was higher (range = 58%–88%) than the percentage among those aged <5 years (range = 18%–45%).

**TABLE:** Percentage of reported respiratory syncytial virus (RSV) cases, by patient age group and polymerase chain reaction (PCR) test positivity — Arizona, 2013–14 through 2016–17 RSV seasons

RSV season	Age group (yrs)					p-value*
	<5	5–14	15–64	≥65	Total	
**2013–14**						
Total no. of cases (%)	2,466 (89)	84 (3)	105 (4)	100 (4)	2,735 (100)	<0.001
No. (%) with PCR-positive tests	446 (18)	36 (43)	47 (45)	58 (58)	587 (21)	
**2014–15**						
Total no. of cases (%)	4,334 (84)	242 (5)	277 (5)	299 (6)	5,152 (100)	<0.001
No. (%) with PCR-positive tests	1,640 (38)	157 (65)	217 (78)	231 (77)	2,245 (44)	
**2015–16**						
Total no. of cases (%)	3,592 (82)	135 (3)	286 (6)	420 (9)	4,433 (100)	<0.001
No. (%) with PCR-positive tests	1,469 (41)	112 (83)	234 (82)	364 (87)	2,179 (49)	
**2016–17**						
Total no. of cases (%)	4,221 (78)	219 (4)	403 (7)	591 (11)	5,434 (100)	<0.001
No. (%) with PCR-positive tests	1,880 (45)	166 (76)	332 (83)	519 (88)	2,897 (53)	

* Chi-squared test for trend.

This shift toward an overall increasing percentage of cases with reported PCR detections since 2013 corresponds with the noted shift in age distribution among reported RSV cases. Although historically RSV has been diagnosed primarily in young children, in recent years, awareness of infection in older adults has increased, possibly reflected by the increase in observed testing in this age group. PCR use differs across age groups, suggesting that the change in age distribution might be attributed to changes in testing practices rather than to changes in the epidemiology of the disease, particularly if there is increased use of PCR-based respiratory viral panels among older adults, who might otherwise not have been tested for RSV. RSV antigen testing is less sensitive in older age groups (*3*), which might further encourage health care providers to order PCR tests instead of antigen tests for older adults.

Because Arizona surveillance data only include positive test results, it was not possible to rule out an age-related change in disease incidence. In addition, the percentage of test results categorized by test type has increased in recent seasons, perhaps as a result of increasing use of electronic laboratory reporting, which facilitates the reporting and entry of more specific test type information, compared with handwritten reports or manual data entry. Future analyses of RSV reporting will include examination of other sources of testing data that include both positive and negative results. As new tools for diagnosing and preventing RSV infection are developed, it is important to understand epidemiologic changes identified through population-based RSV surveillance (*4*).
